# A Hierarchical Framework for Selecting Reference Measures for the Analytical Validation of Sensor-Based Digital Health Technologies

**DOI:** 10.2196/58956

**Published:** 2025-02-07

**Authors:** Jessie P Bakker, Samantha J McClenahan, Piper Fromy, Simon Turner, Barry T Peterson, Benjamin Vandendriessche, Jennifer C Goldsack

**Affiliations:** 1 Digital Medicine Society Boston, MA United States; 2 Department of Electrical, Computer, and Systems Engineering Case Western Reserve University Cleveland, OH United States

**Keywords:** digital health technologies, analytical validation, digital medicine, reference measures, fit-for-purpose digital clinical measures

## Abstract

Sensor-based digital health technologies (sDHTs) are increasingly used to support scientific and clinical decision-making. The digital clinical measures they generate offer enormous benefits, including providing more patient-relevant data, improving patient access, reducing costs, and driving inclusion across health care ecosystems. Scientific best practices and regulatory guidance now provide clear direction to investigators seeking to evaluate sDHTs for use in different contexts. However, the quality of the evidence reported for analytical validation of sDHTs—evaluation of algorithms converting sample-level sensor data into a measure that is clinically interpretable—is inconsistent and too often insufficient to support a particular digital measure as fit-for-purpose. We propose a hierarchical framework to address challenges related to selecting the most appropriate reference measure for conducting analytical validation and codify best practices and an approach that will help capture the greatest value of sDHTs for public health, patient care, and medical product development.

## Introduction

The proliferation of sensor-based digital health technologies (sDHTs) in clinical research and health care delivery has been bolstered by the recent finalization of regulatory guidance supporting their use for remote data acquisitions in clinical investigations [1], qualification of the first sDHT-derived clinical trial endpoint by the European Medicines Agency [2], qualification of the first sDHT-derived medical device development tool by the US Food and Drug Administration (FDA) [3], and the expansion of reimbursement pathways for the use of digital clinical measures in remote patient monitoring [4-6]. For an sDHT to be used to support scientific and clinical decision-making, evaluators must conduct verification of the sensors, usability validation of the user interface (defined as all points of contact a user may have with the sDHT as a whole), analytical validation of the algorithms, and clinical validation of the measures of behavioral or physiological function generated by the sDHT in the proposed context of use. This evaluation process has been codified in the modular V3+ framework by the Digital Medicine Society (DiMe). Since the publication of V3 in 2020 [7] and its extension in 2024 [8], V3+ has become the international methodological standard for sDHT evaluation, having been adopted by dozens of organizations [9] and cited over 250 times [10], including by the European Medicines Agency [11] and FDA [12].

Analytical validation relies on the selection of an appropriate reference measure representing “truth,” against which to compare the output of the sDHT algorithm in the same or directly comparable units. This selection process can be challenging, given that the digital clinical measure of interest may relate to biological and physiological variables, symptoms status, or functional status according to the Wilson and Cleary (1995) [13] conceptual model. In some cases, there may be multiple potential reference measures available, and in the case of a novel digital clinical measure, there will be no extant reference measure at all. We therefore recognize the need for an evidence-based framework to guide decision-making when identifying an appropriate reference measure during analytical validation. The original description of V3 emphasized that not all potential reference measures are of equal quality [7], and as such we believe that a hierarchical step-by-step approach should be adopted to prioritize those representing the highest level of scientific rigor.

We developed a preliminary version of a hierarchical framework to support selection of a reference measure for analytical validation and presented it to experts representing regulators, health technology assessment organizations, sDHT developers, clinicians, and clinical researchers during a workshop convened by the Digital Health Measurement Collaborative Community (DATAcc by DiMe) in November 2023 [14]. Our goal was to seek feedback to improve the framework and ensure uptake across the digital medicine community. Based on this feedback, we describe our hierarchical framework that closes a key gap in the science underpinning the analytical validation of sDHTs.

## Description of the Hierarchical Framework

**Figure 1:**
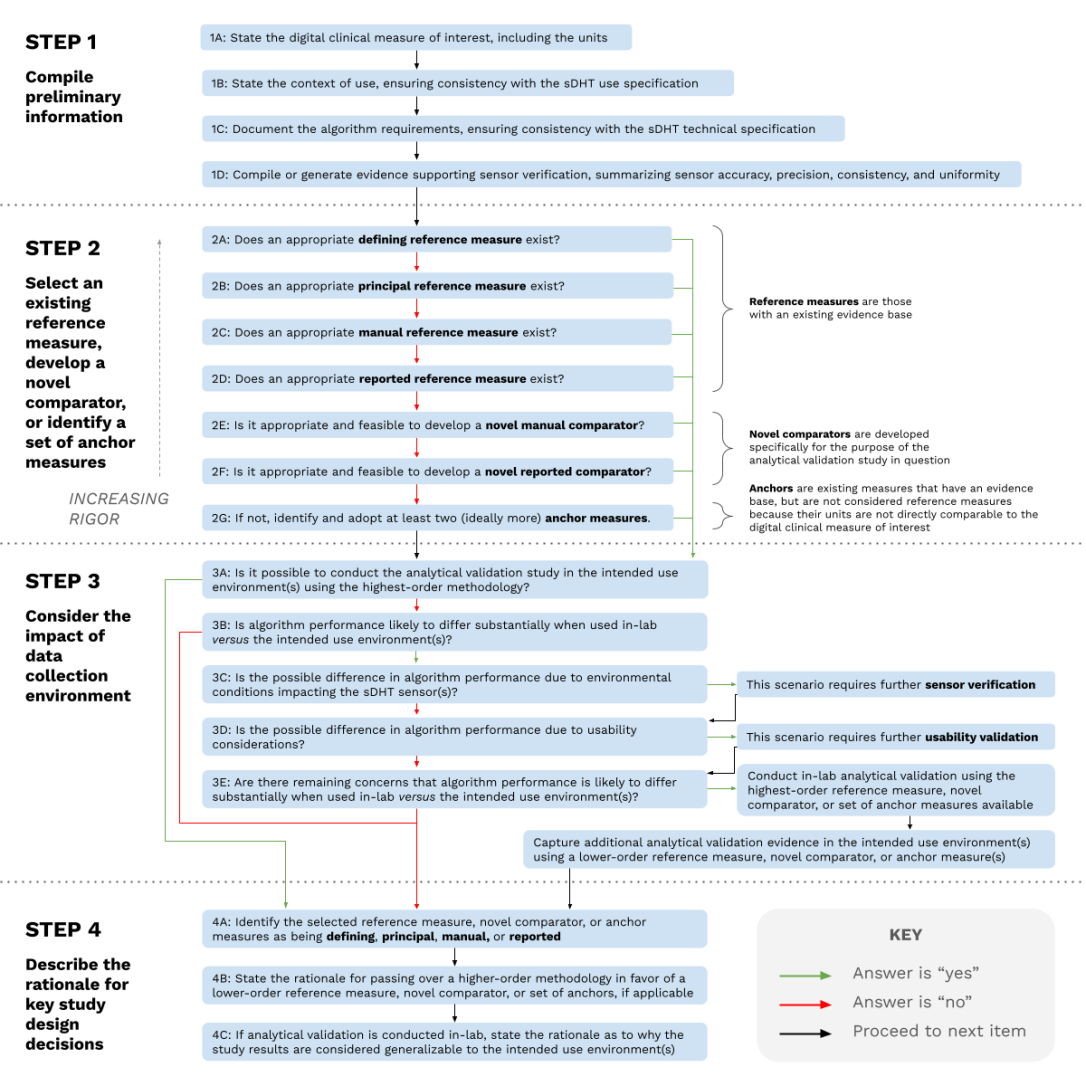
The hierarchical framework for selecting reference measures for analytical validation.

Our framework is designed to sequentially move an investigator or developer through a series of steps laid out in Figure 1 to ensure that the most rigorous reference measures applicable to their study objectives and proposed context of use are prioritized.

### Step 1: Compile Preliminary Information

Step 1 guides the investigator to compile the preliminary information required to determine their analytical validation study objectives and guides subsequent steps. First (Step 1A), the digital clinical measure (a physiologic process or behavioral construct) is described, including the units. Next (Step 1B), the proposed context of use is clarified. The context of use fully and clearly describes the way the sDHT is to be used and the purpose of the use, including the intended populations of interest and the intended use environments. Although some sDHTs may be legally marketed medical devices with an indications for use statement compiled by the technology developer and endorsed by a regulatory body, here we are referring to the context of use proposed by the stakeholder undertaking the analytical validation study. Finally (Steps 1C-1D), the requirements and specifications of the sDHT algorithm are documented in detail, which should include any methods for dealing with missing data, alongside a summary of the verification evidence for each sensor that will feed data to the subject algorithm.

### Step 2: Select an Existing Reference Measure, Develop a Novel Comparator, or Identify Multiple Anchor Measures

Step 2 of the framework classifies potential comparator methods hierarchically according to certain attributes that contribute to their scientific rigor (see Table 1). The first 4 categories represent existing reference measures, while the next 2 categories are required for scenarios in which a reference measure does not yet exist. We describe the latter as novel comparators, because their suitability as an established reference measure cannot be ascertained until they are developed and evaluated. The seventh and final category describes anchor measures, which may be adopted when it is not possible to develop a novel comparator. Unlike reference measures and novel comparators, anchor measures generate data in units that are not directly comparable to those of the digital clinical measure of interest, and therefore analysis is limited to examining associations.

**Table 1:** Hierarchical categorization of reference measures, novel comparators, and anchor measures for analytical validation.

Category		Definition	Attributes	Examples	Rationale for position in the hierarchy	Supporting documentation
**Reference measures**						
	Defining	A defining reference measure emerges when a physiologic process or behavioral construct is dependent on the technology used to capture it to such an extent that it sets the medical definition for that process or construct [15]	Data capture is objective, meaning that it does not rely on human measurement, observation, or perception for acquisition. In some cases, however, human analysis or scoring may be required to generate the measure of interest. It is always possible to retain the source data	The medical definition of sleep staging refers to electrical activity of the brain (EEG), eyes (EOG), and muscles (EMG), as described by the American Academy of Sleep Medicine in the *Manual for the Scoring of Sleep and Associated Events* [16,17]. As such, polysomnography is considered the defining reference for measures of sleep staging, such as total sleep time	Defining and principal reference measures share the same attributes; however, defining references are considered superior as they will always have an associated standards document or equivalent	Standards or guidelines developed by a respected professional body describing the reference measure methodology
	Principal	A principal reference measure directly and objectively measures the physiologic process or behavioral construct of interest	Data capture is objective, meaning that it does not rely on human measurement, observation, or perception for acquisition. In some cases, however, human analysis or scoring may be required to generate the measure of interest. It is always possible to retain the source data	Capnography is considered the defining reference measure for respiratory rate [18], while placement of a nasal pressure transducer, oronasal thermistry, and respiratory inductance plethysmography are considered principal reference measures, as each involves objective data acquisition and the ability to retain the source data	Principal reference measures are considered superior to manual reference measures because the objective method of data capture is not prone to observer bias. Further, the source data can be re-analyzed at will and re-scored by multiple raters (where applicable), thereby promoting standardization and minimizing measurement variability	Descriptions of the reference measure from the peer-reviewed literature, with evidence of standardized implementation in many laboratories and centers*. Or* Standards or guidelines developed by a respected professional body describing the reference measure methodology (in cases where a defining reference measure already exists)
	Manual	A manual reference measure relies on the measurement, observation, or perception of a physiological process or behavioral construct by a trained health care professional, with or without the use of equipment or technology	The measure of interest can be seen, heard, or felt; taste and smell have been relied on historically but are not routinely used in modern medicine. Although it may be possible to retain source data (such as a video that is then manually annotated), many manual reference measures are made in real time	Respiratory rate may be captured by auscultation or by visually assessing chest wall movement [19]	Manual reference measures are considered superior to reported reference measures because they are made by trained health care professionals which promotes standardization. In some cases it may also be possible to obtain manual measures from multiple raters, thereby reducing measurement variability	Descriptions of the reference measure from the peer-reviewed literature, with evidence of standardized implementation in many laboratories and centers. *Or* Standards or guidelines developed by a respected professional body describing the reference measure methodology (in cases where a defining reference measure already exists)
	Reported	A patient-reported reference measure is based on a report that comes directly from a patient about the status of their health condition, while an observer-reported reference measure is based on a report from another individual based on observable signs, events, or behaviors related to a patient’s health condition [20]	The identification or quantification of the measure is subjective in nature. Reports are made either in real time or retrospectively	The presence or absence, frequency, or duration of a particular experience or event is typically captured through diaries; for example, use of the Consensus Sleep Diary to capture time in bed [21]	Reported reference measures are considered inferior to manual reference measures because they typically involve a high degree of subjectivity or interpretation, and each measure can be generated only once per timepoint	Peer-reviewed evidence of psychometric performance of the instrument used for data capture
**Novel comparators**						
	Manual	If the digital clinical measure of interest is based on a characteristic that can be observed or perceived by a trained health care professional, it may be possible to develop a novel manual comparator	A novel manual comparator shares the attributes of a manual reference measure described earlier	Manual annotation of a video captured overnight to identify measures of nocturnal scratch [22,23]	See rationale for manual reference measures described earlier	Study protocol, including all data acquisition, data processing, and scoring or annotation methods, developed *a priori*. We recommend collaborating with patient representatives when developing a definition for the characteristic of interest
	Reported	If the digital clinical measure cannot be observed or perceived by a trained health care professional and is instead best captured by a report from the patient themselves or by an observer, it may be possible to develop a novel reported comparator	A novel reported comparator shares the attributes of a reported reference measure described earlier	Self-report of the time intervals for daily activities in a diary developed for the purpose of analytical validation [24]	See rationale for reported reference measures described earlier	Peer-reviewed publication, study report, white paper containing evidence supporting face validity. As described earlier, we recommend collaborating with patient representatives when developing the novel reported comparator
**Anchor measures**						
	Anchors	An anchor measure is any interpretable measure of a physiologic process or behavioral construct in units that are not directly comparable to the digital clinical measure of interest. Units considered directly comparable are either identical or able to be translated for the purposes of comparison, such as via calibration	Anchor measures may possess any of the attributes listed earlier for defining or principal, manual, or reported reference measures	MDS-UPDRS Motor Assessment Item 3.15 involves a trained health care professional evaluating Parkinsonian postural tremor of the hands by visually determining tremor amplitude, returning a score of 0-4 [25].This score is not directly comparable to a digital clinical measure of tremor amplitude in units of acceleration; as such, it should be used alongside at least one more anchor measure of tremor severity, such as the Patient-Reported Outcomes in Parkinson’s Disease rating scale [26]. Readers should note that in this particular example, a reference measure (3D motion capture) already exists	Anchor measures are not directly comparable to the digital clinical measure of interest, and therefore analysis is limited to examining associations. Unlike reference measures and novel comparators, anchors are not suitable for analytical validation in isolation; instead, multiple anchors should be identified in order to strengthen the assertion that the algorithm measures what it purports to measure	Supporting documents may be in the form of anything listed earlier for defining or principal, manual, or reported reference measures

The highest position of the hierarchy is assigned to *defining* reference measures (Step 2A), which are objective measures that emerge when the medical definition of a clinical measure is dependent on the technology used to capture it [15]. These measures do not rely on human observation or perception for data capture, although in some cases human analysis or scoring may be required to generate the measure of interest. Defining references are those that are widely accepted, endorsed by at least one acclaimed professional body, and described within a standards document or equivalent. For example, the American Academy of Sleep Medicine describes polysomnography (the measurement of brain, eye, and muscle activity through electroencephalography, electrooculography, and electromyography, respectively) as the defining method for capturing sleep staging data [16,17]. Similarly, the International Organization for Standardization (ISO) identifies CO-oximetry, based on light absorption of arterial blood samples typically obtained during hypoxia challenge, as the defining method for measuring blood oxygen saturation [27].

The next highest level of rigor is assigned to *principal* reference measures (Step 2B), which share the attributes of defining reference measures but typically do not have a dedicated standards document; instead, there are in-depth descriptions of the methodology in the peer-reviewed literature along with evidence of standardized implementation in many laboratories and centers. In some cases, a reference measure that does have an associated standards document may be considered a principal reference measure because a superior defining reference measure also exists. Examples of the latter scenario include transmissive and reflectance pulse oximetry; although data capture is objective and the methodology is referred to in both ISO 80601-2-61:2017 and regulatory guidance [27,28], these methods are considered inferior to CO-oximetry (the defining reference measure), because they are not capable of distinguishing functional and nonfunctional hemoglobin [29]. As this example illustrates, there may be more than one principal reference measure available to choose from—unlike defining reference measures, of which there can only be one—each of which may perform differently depending on the context of use.

Next, *manual* reference measures (Step 2C) rely on observation or perception by a trained health care professional in the absence of a source of objectively acquired data. Manual reference measures may or may not be aided by equipment or technology; for instance, respiratory rate can be measured manually through visual inspection of breaths or through auditory interpretation of auscultation using a stethoscope.

Next, *reported* reference measures (Step 2D) are based on reports that come directly from a patient (study participant) about the status of their health or a report from a lay observer such as a parent or care partner, using data capture instruments supported by appropriate psychometric evaluation. We have based our definition of reported reference measures on the BEST resource definitions of patient- and observer-reported outcomes [20]; however, it is important to note that many patient-reported outcomes and observer-reported outcomes are generated using questionnaires that are not suitable as reference measures because they do not adopt units that are directly comparable to those of the digital clinical measure of interest. For example, sDHT-derived nocturnal scratch in units of events/hour cannot be directly compared against the Patient-Oriented Eczema Measure, which generates a score of 0-28 [30]; instead, investigators have relied on annotated videos [22]. Reported reference measures are therefore limited to reports of the presence or absence, frequency, or duration of a particular experience or event, typically captured using diaries supported by appropriate psychometric evaluation.

If no suitable reference measures exist, the investigator should consider developing a novel comparator, which should be completed and documented before beginning analytical validation. When developing a *novel manual comparator* (Step 2E), which shares the attributes of a manual reference measure, the investigator should develop a protocol for capturing and analyzing the comparator measure *a priori*, using existing technology where applicable. If it is not possible to create a novel manual comparator, the investigator should consider the development of a *novel reported comparator* (Step 2F), which shares the attributes of a reported reference measure and should be supported by evidence of face validity (at minimum). We recommend working with patient representatives when developing novel manual or reported comparators.

If it is not possible to develop a novel comparator, the only remaining option is to adopt *anchor measures* (Step 2G), which are defined as any interpretable measures of a physiologic process or behavioral construct in units that are not directly comparable to the digital clinical measure of interest. This broad definition means that an anchor measure may share attributes with defining, principal, manual, or reported reference measures. Because the data generated by an anchor measure cannot be directly compared to the digital clinical measure, it is critical to adopt multiple anchors in order to strengthen the assertion that the algorithm measures what it purports to measure. The use of multiple anchor measures for analytical validation purposes aligns with the concept of an anchor variable described by the FDA in their Patient-Focused Drug Development guidance for identifying meaningful score differences of clinical outcome assessments [31].

### Step 3: Consider the Impact of the Data Collection Environment

Step 3 of the framework guides the investigator through a series of questions regarding the impact of the data collection environment. First (Step 3A), the investigator should determine whether it is possible to conduct analytical validation in the intended use environments using the highest-order reference measure, novel comparator, or anchor measures available. If so, they should plan to do so and proceed to Step 4.

If the highest-order reference measure, novel comparator, or anchor measures require the analytical validation study to be conducted in the laboratory, the investigator should determine whether the performance of the algorithm is likely to differ substantially when the sDHT is implemented in the intended use environments (Step 3B). If this is not the case and the investigator determines that algorithm performance assessed in the laboratory is generalizable to the intended use environment, they should complete the analytical validation study in the laboratory and proceed to Step 4.

Alternatively, if the investigator is concerned with generalizing analytical validation results from the laboratory setting to the intended use environments, they should first determine whether the possible difference in algorithm performance is due to environmental conditions impacting the sDHT sensors (Step 3C). If so, this scenario requires additional verification data and is not a reason to perform analytical validation in the intended use environment. Next, the investigator should determine whether the possible difference in algorithm performance is due to usability considerations (Step 3D); for example, they may be concerned that participants will place the sDHT incorrectly when using it at home unsupervised, leading to excessive signal artifact. If so, this scenario requires usability validation, and is similarly not a reason to perform analytical validation in the home environment.

Finally, if the investigator determines that the possible difference in algorithm performance is due to environmental characteristics impacting the relationship between algorithm input (sensor data) and output (the digital clinical measures of interest) (Step 3D), the investigator should proceed with in-laboratory analytical validation using the highest-order reference measure, novel comparator, or anchor measures, and plan to capture additional analytical validation evidence in the intended use environments using a lower order alternative. In other words, the investigator should first determine algorithm performance using the most rigorous methodology available and perform supplementary testing to support generalizability to the intended use environments, rather than relying solely on the lower order methodology. It is acceptable for supplementary evidence of this nature to rely on a single anchor measure, if applicable, although multiple anchors are preferable.

### Step 4: Describe the Rationale for Key Study Design Decisions

The final step of the framework prompts the investigator to describe their rationale for key study design decisions by identifying their selected reference measure, novel comparator, or anchor measures according to the categories described in Step 2 of this framework (Step 4A); provide a rationale for passing over a higher order methodology in favor of their selected methodology, if applicable (Step 4B); and, if the analytical validation study is conducted in the laboratory, provide a rationale as to the generalizability of their results to the intended use environments. Step 4 is particularly important, as it encourages transparency and allows evaluators, peer-reviewers, regulators, and payers to understand the quality of the resulting analytical validation data and the extent to which it may be relied upon for decision-making.

## Discussion

### The Scientific Rigor of Reference Measures Is Driven by Attributes That Reduce Measurement Variability

The categories of reference measures in Step 2 are laid out in order of superiority to signify that the highest-ranked reference measure that is available should be used whenever possible, as according to the definitions in Table 1, it is the best method for capturing the measure of interest. Our goal was to rank the categories according to attributes that contribute to reduced measurement variability.

Both *defining* and *principal* reference measures rely on objective methods of data acquisition, and the ability to retain source data means that these reference measures can be re-analyzed or re-scored at will, including by multiple health care professionals when applicable in order to create an average or consensus measure, thereby reducing measurement variability [32]. The next category, *manual* reference measures, involves human perception and is therefore prone to observer bias, but likely not to the extent of *reported* reference measures, as these typically involve a high degree of subjectivity or interpretation, and each measure can be generated only once per timepoint. Although we champion the importance of the voice of the patient during the selection of measures that matter to scientific and clinical decision-making [33-36], it is expected that the processes of usability and clinical validation will sufficiently assess these important concerns, which are out of scope for analytical validation.

We have positioned anchor measures as the lowest-order methodology, even if an anchor under consideration is well-established and widely used as illustrated by the example in Table 1 describing a comparison of sDHT-derived postural tremor (in units of acceleration) against MDS-UPDRS Motor Assessment Item 3.15 (in units of a 0-4 score). If this scale were to be adopted as the sole anchor measure, the developer would be motivated to create an sDHT algorithm that aligns as closely as possible with the clinician’s visual assessment of tremor amplitude, thereby perpetuating the known limitations of this methodology. Thus, it is essential that multiple anchors be adopted for the purpose of evaluating a novel digital clinical measure, although under some circumstances it may be sufficient to adopt a single anchor if the intent is to generate supplementary evidence to support the generalizability of more robust analytical validation results from the laboratory to the intended use environments. Readers should note that comparison of a digital clinical measure against anchor measures is not a form of analytical validation given the noncomparable units but is a suitable approach to addressing this component of V3+ when reference measures do not exist and it is not possible or feasible to develop a novel comparator.

### Circumstances Under Which a Lower Ranked Reference Measure May Be Appropriate for Analytical Validation of an sDHT

Our hierarchical framework will enable investigators conducting analytical validation to make the most rigorous claims associated with their products and position them to deliver the greatest value in support of scientific and clinical decision-making. Importantly, however, the hierarchical nature of Step 2 is not meant to be rigid. In some circumstances, there may be reasons for passing over a reference measure of the highest rigor to select a reference measure or novel comparator that is ranked lower. However, we posit that the only acceptable reasons for doing so are when (1) the selection of the higher ranked reference measure creates an unacceptable risk-benefit ratio, thereby compromising the ethics of the study or (2) the higher ranked reference measure is not recommended and applicable to the context of use, which includes the patient population.

Consider, for example, the analytical validation of an sDHT developed to capture blood pressure (BP). The *defining* reference measure involves placement of an arterial catheter [37], which is invasive and in some circumstances may be considered unnecessarily risky to participants. Because automated sphygmomanometry (a *principal* reference measure) is such a common and well-validated technology, it may be appropriate to use in place of arterial BP in some cases. Automatic sphygmomanometers, however, may not be suitable for use in some individuals, such as those with arrhythmias [38], in which case traditional sphygmomanometry (a *manual* reference measure) may be the most appropriate methodology. An investigator may select any reference measure or novel comparator of their choosing, with the understanding that adoption of a lower order methodology will limit the rigor of their analytical validation study.

It is rarely a requirement to select a reference measure that allows analytical validation to be conducted in remote settings, such as at home, particularly if this choice would result in the use of a reference measure that is lower in the hierarchy. However, when analytical validation is conducted in the laboratory, it is important to determine whether the performance of the algorithm is likely to differ substantially when the sDHT is implemented in its intended use environments as described in Step 3.

In summary, it is up to the investigator or developer to justify their choice by carefully considering the scientific or clinical decisions that the sDHT-derived measure is intended to support and the rigor of the analytical validation claims they wish to make (Step 4). Factors such as cost, feasibility, patient or participant preference, and resource availability are rarely sufficient reasons to select a lower order reference measure or novel comparator but might be relevant to investigators seeking to decide between reference measures within a single category.

### The Quality of the Reference Measure or Novel Comparator Affects What Claims Can Be Made About the Performance of the sDHT

A key value driver of sDHTs is their ability to capture digital clinical measures that provide information that was previously difficult or impossible to capture, such as the measurement of nocturnal scratch in individuals with atopic dermatitis [39]. In such cases, available reference measures for analytical validation are likely to be ranked lower per the framework if they are available at all. The development and use of a novel comparator does not preclude the substantiation of rigorous claims associated with these measures but does require careful development of the novel comparator *a priori*. Over time, a novel comparator may “up-level” to be considered a reference measure as evidence to support its use builds over time. Manual annotation of video to identify nocturnal scratch events, even if performed by multiple trained health care professionals, can currently be considered a novel manual comparator despite being adopted in more than one study [22,23]. Eventually, assuming this line of work continues, the field may coalesce around a video scoring protocol that allows for a high level of interrater agreement, at which point the method may be considered a manual reference measure.

### Applicability of the Framework to All sDHT Regulatory Categories and All Measure Categories

As described in regulatory guidance and the literature [1,40], sDHTs used for remote data acquisition in clinical investigations may be regulated medical devices, low-risk products designed to promote a healthy lifestyle and typically marketed towards consumers (referred to by the FDA as “general wellness products” [41]), or products developed specifically for research data capture. Although the term “claims” may be used to refer to statements that fall under regulatory purview, such as medical device labeling claims, throughout this document we use “claims” to refer to any statement or conclusion made regarding the performance of an sDHT. The principles of analytical validation apply in all cases, and therefore the process of selecting a reference measure or novel comparator does not differ according to the regulatory status of the sDHT.

Similarly, the principles of analytical validation do not differ according to whether the digital clinical measure of interest is intended for use in clinical care or clinical research. If the latter, the categorization of the measure as a digital biomarker or an electronic clinical outcome assessment also has no bearing on the applicability of our framework.

### Future Directions

During development of this framework, we considered the suitability of a scoring method that would allow an investigator to rank reference measures quantitatively, rather than qualitatively according to the categories described in Step 2. Ultimately, we decided against this approach due to concerns that a scoring system would be unnecessarily rigid and may quickly become outdated as technology evolves. As the field matures, however, a quantitative scoring system may be warranted.

The hierarchical framework presented here will help investigators support the most rigorous claims for both novel and established digital clinical measures and is intended to drive the capture of the greatest value of sDHTs, whether from their use in clinical trials [42] or patient care [43]. As the selection of reference measures or development of novel comparators for the analytical validation of sDHT algorithms becomes increasingly rigorous through the use of this framework, it is important to highlight a key remaining gap in the science of analytical validation: the selection of a suitable statistical technique for evaluating an algorithm's performance against the appropriate reference measure. Despite this remaining gap, the state of the science supporting the evaluation of sDHTs as fit-for-purpose is strong. This framework offers a pathway to bolster the impact of the broad adoption of best practices, such as the Measures that Matter [44] and V3+ frameworks [7,8], alongside advances in regulatory policies and decisions [1,2], placing the benefits of digital clinical measurement firmly within the reach of both researchers and clinicians seeking to improve the health-related quality of life of their patients. Ultimately, the value of our proposed framework will be determined according to its impact on improving the rigor of sDHT analytical validation science.

## Abbreviations

BP: blood pressure
DiMe: Digital Medicine Society
FDA: Food and Drug Administration
ISO: International Organization for Standardization
sDHT: sensor-based digital health technology
